# Pores for Thought: Can Genetic Manipulation of Stomatal Density Protect Future Rice Yields?

**DOI:** 10.3389/fpls.2019.01783

**Published:** 2020-02-11

**Authors:** Christopher R. Buckley, Robert S. Caine, Julie E. Gray

**Affiliations:** Department of Molecular Biology and Biotechnology, University of Sheffield, Sheffield, United Kingdom

**Keywords:** stomatal development, rice, crop yields, stomatal density, grasses, crop physiology

## Abstract

Rice (*Oryza sativa* L.) contributes to the diets of around 3.5 billion people every day and is consumed more than any other plant. Alarmingly, climate predictions suggest that the frequency of severe drought and high-temperature events will increase, and this is set to threaten the global rice supply. In this review, we consider whether water or heat stresses in crops — especially rice — could be mitigated through alterations to stomata; minute pores on the plant epidermis that permit carbon acquisition and regulate water loss. In the short-term, water loss is controlled *via* alterations to the degree of stomatal “openness”, or, in the longer-term, by altering the number (or density) of stomata that form. A range of molecular components contribute to the regulation of stomatal density, including transcription factors, plasma membrane-associated proteins and intercellular and extracellular signaling molecules. Much of our existing knowledge relating to stomatal development comes from research conducted on the model plant, *Arabidopsis thaliana*. However, due to the importance of cereal crops to global food supply, studies on grass stomata have expanded in recent years, with molecular-level discoveries underscoring several divergent developmental and morphological features. Cultivation of rice is particularly water-intensive, and there is interest in developing varieties that require less water yet still maintain grain yields. This could be achieved by manipulating stomatal development; a crop with fewer stomata might be more conservative in its water use and therefore more capable of surviving periods of water stress. However, decreasing stomatal density might restrict the rate of CO_2_ uptake and reduce the extent of evaporative cooling, potentially leading to detrimental effects on yields. Thus, the extent to which crop yields in the future climate will be affected by increasing or decreasing stomatal density should be determined. Here, our current understanding of the regulation of stomatal development is summarised, focusing particularly on the genetic mechanisms that have recently been described for rice and other grasses. Application of this knowledge toward the creation of “climate-ready” rice is discussed, with attention drawn to the lesser-studied molecular elements whose contributions to the complexity of grass stomatal development must be understood to advance efforts.

## Introduction

Feeding a global population expected to reach 9.8 billion by 2050 is one of the greatest challenges of our time (McGuire, 2015; United Nations, 2017). Together with rising temperatures and dwindling reserves of natural resources such as freshwater, the problem is exasperated by growing climate instabilities (Stott, 2016). The frequency and intensity of extreme weather events are projected to increase under future climate scenarios, which will likely lead to substantial losses of crop yields (Seneviratne et al., 2012; Challinor et al., 2014). Thus, to alleviate the risk of crop failures, the development of “climate-ready” crops that can withstand future climatic stresses should be prioritized.

Rice (*Oryza sativa* L.) is a major source of food and income for billions worldwide. In Asia, where over 90% of rice is grown and consumed (Brar and Khush, 2013; Elert, 2014), the primary climatic factor for the majority of the growing region is the monsoon, which contributes >80% of annual rainfall within a few months (Haefele et al., 2016). Rainfed cultivation accounts for ~20% of global rice supply yet, as a consequence of this irregular rainfall, productivity in rainfed regions is already constrained by drought (Wassmann et al., 2009; Maclean et al., 2013; Swain et al., 2017). Rice is also susceptible to heat stress, particularly during the reproductive and grain filling stages (Matsui et al., 2000; Lin et al., 2010), and there are concerns that both high-temperature events and droughts are expected to become more prevalent across the world’s rice cultivation regions (Krishnan et al., 2011). Furthermore, there is an increasing requirement to limit water use for crop irrigation, which already accounts for around 70% of global freshwater usage (Foley et al., 2011). It is therefore important that water use efficiency (WUE; defined here as the amount of carbon assimilated per unit of water lost) and tolerances to heat-stress and drought are improved to ready rice for future climates.

Plants use microscopic epidermal openings called stomata to regulate the uptake of CO_2_ and the release of water between internal tissue surfaces and the environment (Buckley, 2005). Typically consisting of a pair of specialised turgor-driven guard cells (GCs) surrounding a central pore, stomata also function to regulate plant temperature and to move solutes and water internally *via* the transpiration stream. Under water-limiting conditions stomata close, thereby restricting water loss *via* reduced stomatal conductance (*g_s_*), with CO_2_ uptake for photosynthesis (*A*), evaporative cooling, and nutrient transfer often diminished as a result (Arve et al., 2011; Urban et al., 2017). Because less cooling occurs, stomatal closure exacerbates the risk of plant organs reaching lethally high temperatures which, particularly for rice in lower latitudes, may lead to substantial crop losses (Long and Ort, 2010; Sánchez et al., 2014).

Adjustments to the size of the stomatal pore permit a near-immediate plant response to a change in the surrounding environment (Brownlee, 2001). However, when sustained environmental stimuli arise over longer durations, alterations to stomatal density (SD; the number of stomata that form per unit area) and size may also occur (Casson and Gray, 2008; Qi and Torii, 2018). By increasing SD, *g_s_* might be increased, which in turn may lead to greater evaporative cooling and increases in *A* (Tanaka et al., 2013). Conversely, by reducing SD, *g_s_* can be lowered, which may curb water loss and result in improved WUE and drought tolerance (Doheny-Adams et al., 2012; Caine et al., 2019). If reductions in SD do not have a significant detrimental impact on *A* or evaporative cooling, the overall benefits of increased water conservation could represent a viable strategy to protect certain crops, including rice, against future extreme weather events (Hepworth et al., 2018).

A deep understanding of the stomatal development program in rice is required for genetic manipulation of SD. The majority of our existing knowledge stems from the model plant Arabidopsis (*Arabidopsis thaliana*), and this is beginning to be translated into grass species (to name a few: Raissig et al., 2016; Raissig et al., 2017; Yin et al., 2017; Abrash et al., 2018; Lu et al., 2019; Mohammed et al., 2019; Wu et al., 2019). In this review, our current understanding of the molecular-level control of stomatal development in rice will be summarized, drawing comparisons and distinctions between Arabidopsis, the model grass Brachypodium (*Brachypodium distachyon*), and other grass species of agricultural importance such as maize (*Zea mays*) and barley (*Hordeum vulgare*). Then, translating this knowledge into action, recent advances towards “climate-ready” rice with altered SD will be discussed.

## Grasses Have Conserved and Divergent Elements of Stomatal Morphology and Development

**Figure 1:**
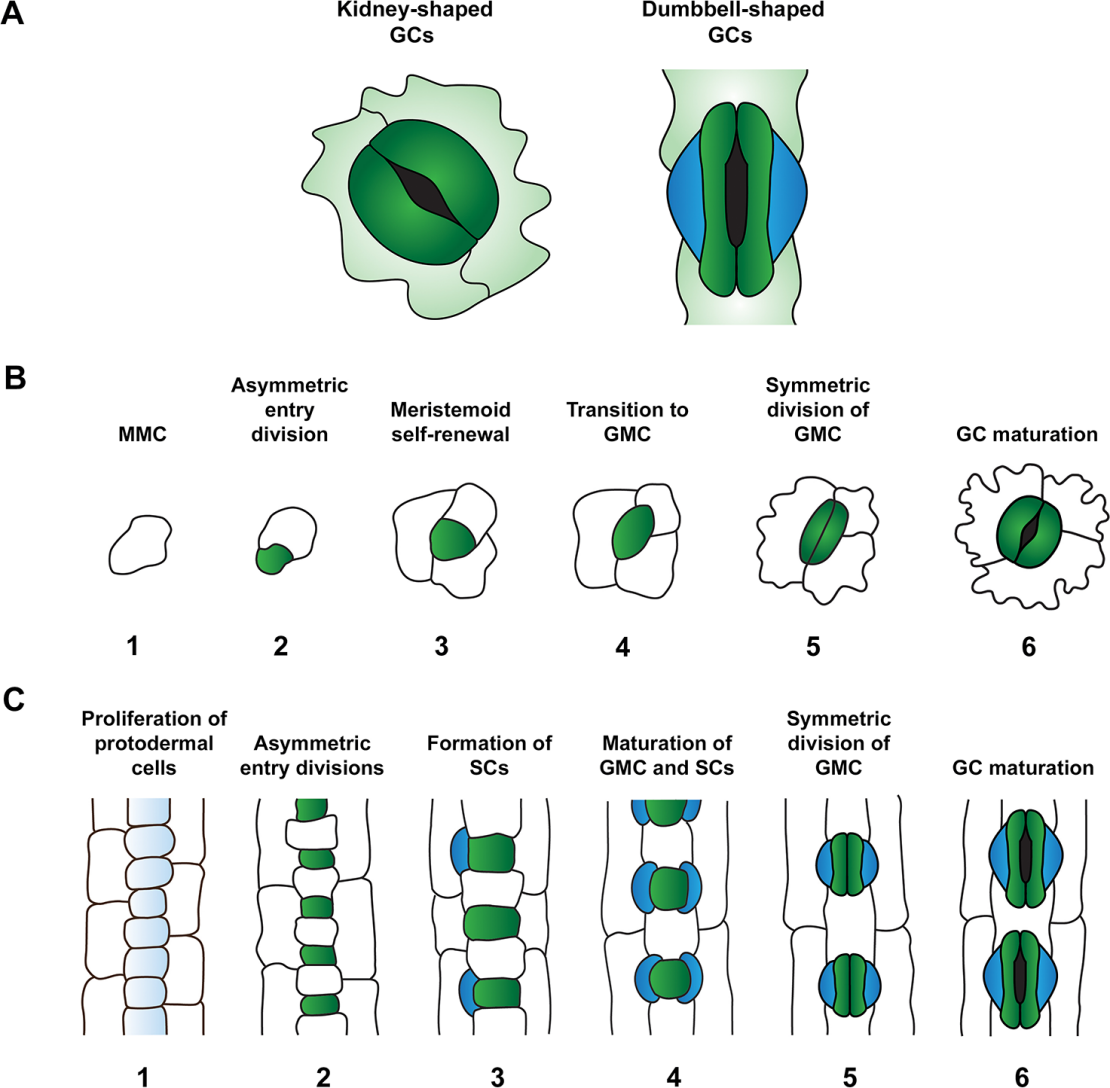
Comparison of stomatal morphology and development in the leaves of the eudicot *Arabidopsis thaliana* and monocot grasses. **(A)** Comparison of guard cell (GC) morphology: kidney-shaped GCs of *A. thaliana* (left) and dumbbell-shaped GCs typical of monocot grasses (right). GCs and subsidiary cells (SCs) are depicted in dark green and blue respectively. **(B)** Stomatal development in *A. thaliana*. Meristemoid mother cells (MMCs) (1) divide asymmetrically to form meristemoids (green) (2). This process of asymmetric division is repeated in the self-renewing meristemoid phase (3). Note, asymmetric spacing divisions of MMCs occur around this developmental stage, with newly formed satellite meristemoids forming at least one-cell away from pre-existing meristemoids and stomata. Asymmetric divisions are then terminated and meristemoids transition to guard mother cell (GMC) state (4). GMCs undergo a single symmetric division to produce a pair of GCs (5) which mature to define the stomatal aperture amid the tessellated pavement cells of the leaf epidermis (6). **(C)** Stomatal development in grasses (modelled from barley, *Hordeum vulgare*). At the beginning of stomatal development, protodermal cells start to proliferate at a faster rate than surrounding cell files (1). Protodermal cells undergo just one round of asymmetric division, generating smaller GMCs (green) and larger epidermal cells (2). GMCs become enlarged and provide cues to induce asymmetric divisions in flanking subsidiary mother cells (SMCs) (3) which result in the production of SCs (blue) (4). Analogous to eudicot stomatal development, GMCs undergo a single symmetric division (5) to produce a pair of daughter cells that differentiate into mature GCs surrounded by columnar pavement cells (6).

Rice and other cereal crops such as maize, barley, and wheat (Triticum aestivum) are monocot grasses and, relative to eudicot plants such as Arabidopsis, are defined by a number of distinctive features relating to where and how stomata develop (Raissig et al., 2016; Conklin et al., 2018; Hepworth et al., 2018). In grass leaves, stomata are organized into evenly distributed parallel files which are interspersed either side of leaf veins, with the origin of stomatal development primarily occurring at the leaf base (Conklin et al., 2018). Whereas stomatal development is primarily initiated at the leaf base in Arabidopsis, the location of development is not as constrained as in grasses and the spatial distribution of stomata appears more random as a result (Conklin et al., 2018). The culmination of stomatal development for grasses is a cellular complex composed of a pair of dumbbell-shaped GCs adjoined on either side by subsidiary cells (SCs) (**Figure 1**). These complexes are typically separated by at least one epidermal cell and thus development follows the one-cell spacing rule (Sachs, 1991; Chen et al., 2017; Hepworth et al., 2018). In Arabidopsis, stomatal GCs are kidney-shaped (**Figure 1**) and are without flanking SCs but, like grasses, the one-cell spacing rule is maintained (Hara et al., 2007).

For most plant species, including Arabidopsis and grasses, the stomatal lineage progresses in much the same way; originating from a set of undifferentiated epidermal cells at the base of the leaf known as protodermal cells (Stebbins and Shah, 1960; Yang et al., 1995; Luo et al., 2012; Pillitteri and Dong, 2013; Raissig et al., 2016; Hepworth et al., 2018; Endo and Torii, 2019). The stomatal lineage is initiated when individual protodermal cells (known in Arabidopsis as meristemoid mother cells, MMCs) gain the capacity to undergo asymmetric cell divisions (**Figure 1**). In both Arabidopsis and grasses an asymmetric entry division leads to the formation of one smaller daughter cell and one larger daughter cell (**Figure 1**). The larger cell in Arabidopsis is termed a stomatal lineage ground cell (SLGC), while in grasses this cell is typically referred to simply as a sister cell or long cell. The smaller cell in Arabidopsis is a meristemoid and retains the ability to undergo self-renewing amplifying divisions, often generating several new SLGCs as a result (**Figure 1**). Although Wu et al. (2019) refer to the smaller cells in grasses as meristemoids, there is no evidence in the literature to suggest that these cells possess the ability to undergo amplifying divisions; instead the smaller daughter cell expands slightly and transitions directly to a guard mother cell (GMC) (Raissig et al., 2016; Hepworth et al., 2018). For clarity, in this review we will refer to the smaller cells derived from grass asymmetric divisions as GMCs. While Arabidopsis SLGCs have the ability to undergo a spacing division to form satellite meristemoids (Pillitteri and Dong, 2013), similar functionality has not been reported for the larger sister cells of grasses. However, since the proximal-distal development of grass stomata is not completely linear, and multiple large epidermal cells can develop adjacently without the presence of an intervening GMC (Hepworth et al., 2018), the possibility that larger sister cells can undergo infrequent satellite divisions to produce new GMCs cannot be ruled out.

The divergent developmental processes described above during the beginning of the stomatal lineage are briefly realigned when meristemoids in Arabidopsis transition to GMCs and take on a form similar to that of grass GMCs (**Figure 1**). However, while Arabidopsis GMCs are typically programmed to divide symmetrically to form a pair of GCs, grass GMC cell state is temporarily maintained (Luo et al., 2012; Pillitteri and Dong, 2013; Hepworth et al., 2018). This allows asymmetric divisions to occur in both of the adjacent subsidiary mother cells (SMCs), resulting in the production of a pair of SCs either side of the GMC. Once SCs have formed, grass GMCs — like Arabidopsis GMCs — undergo a single symmetric division to form a pair of GCs. The termination of stomatal development occurs when nascent Arabidopsis and grass GCs fully differentiate to become kidney-shaped and dumbbell-shaped cells respectively (Conklin et al., 2018) (**Figure 1**).

## Stomatal Lineage Initiation and Advancement is Governed by Transcriptional Regulators

**Figure 2:**
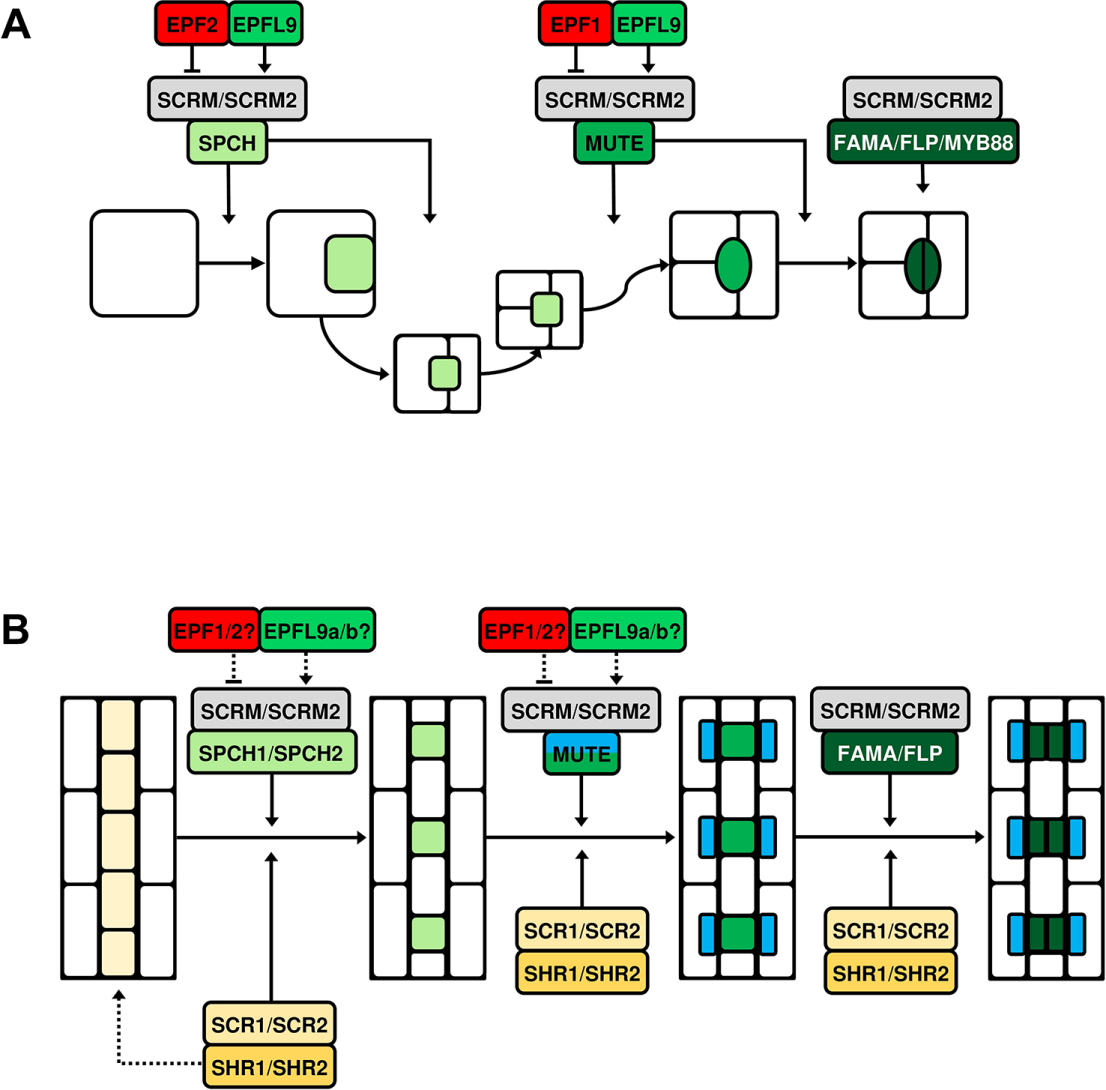
Simplified models of bHLH-mediated stomatal cell lineage transitions in *Arabidopsis thaliana* and grasses. Five basic helix-loop-helix (bHLH) transcription factors, SPEECHLESS (SPCH), MUTE, FAMA, and their heterodimeric partners SCREAM (SCRM) and SCRM2 exhibit control over stomatal cell type transitions and are conserved across land plants. **(A)** Stomatal development in *A. thaliana*. SPCH directs asymmetric divisions of meristemoid mother cells (MMCs) and promotes the meristemoid self-renewing divisions. Epidermal patterning factor 2 (EPF2) and EPF-like protein 9 (EPFL9) are antagonistic regulators of SPCH; competing to suppress and promote its activity respectively. MUTE terminates the meristemoid self-renewing state and promotes guard mother cell (GMC) differentiation and GMC symmetric division. EPF1 primarily targets MUTE; by restraining its activity extra symmetric divisions of GMCs are prevented. EPF1, like EPF2, is assumed to compete with EPFL9 for the regulation of MUTE. FAMA also prevents ectopic symmetric divisions of GMCs and ultimately promotes guard cell (GC) maturation together with the closely related MYB proteins FOUR LIPS (FLP) and MYB88. At each stage of transition, SPCH, MUTE and FAMA form obligate heterodimer complexes with SCRM or SCRM2. **(B)** The molecular control of stomatal development is ‘alternatively wired’ in monocotyledonous grasses such as rice (*Oryza sativa*) and Brachypodium (*Brachypodium distachyon*). It has been suggested that the transcription factors SHORTROOOT1 and SHORTROOT2 (SHR1/2) are involved in the establishment of stomatal lineage cell files (dashed arrow). SPCH has been duplicated and both homologues act to induce asymmetric divisions in these stomatal lineage cells, with SPCH2 the more predominant actor. MUTE promotes GMC differentiation and is involved in the formation of subsidiary cells (SCs) in neighboring subsidiary mother cells (SMCs). As in Arabidopsis, FAMA inhibits supernumerary symmetric divisions of GMCs, probably in combination with a single FLP protein. SCRM and SCRM2 also appear to form heterodimer complexes with the aforementioned bHLHs throughout the lineage, as is observed in Arabidopsis. SHR1/2 and their partner transcription factors SCARECROW1/2 (SCR1/2) exert regulatory influence at each stage of transition. EPFs/EPFLs are also present during grass stomatal development and, while they can demonstrably constrain or promote development, exactly where and how they regulate the grass bHLHs has not been determined (dashed arrows).

In Arabidopsis, five basic helix-loop-helix (bHLH) transcription factors are primarily responsible for regulating stomatal lineage entry and subsequent advancement to mature stomata. These bHLHs are known as SPEECHLESS (SPCH), its close paralogues MUTE and FAMA, and SCREAM (SCRM, also known as ICE1) and SCRM2 (Ohashi-Ito and Bergmann, 2006; MacAlister et al., 2007; Pillitteri et al., 2007; Kanaoka et al., 2008) (**Figure 2**). The formation of MMCs, asymmetric entry divisions and subsequent meristemoid self-renewal requires the first of these, SPCH (Pillitteri and Dong, 2013; Endo and Torii, 2019). MUTE activity is then required to terminate asymmetric divisions and promote the differentiation of meristemoids into GMCs (Pillitteri et al., 2007). Recent work by Han et al. (2018) has shown that MUTE also promotes the GMC-to-GC division by directly inducing the expression of a series of cell-cycle genes that trigger the symmetric divisions of GMCs. MUTE also promotes the transcriptional repressor of these genes, FAMA, which together with the closely related MYB proteins FOUR LIPS (FLP) and MYB88, acts to inhibit extraneous symmetric divisions of GMCs and promote GC maturation (Ohashi-Ito and Bergmann, 2006; Han et al., 2018). At each specific stage of cell division and transition, SPCH, MUTE, and FAMA form obligate heterodimer complexes with SCRM and SCRM2 (Kanaoka et al., 2008; Conklin et al., 2018). These complexes are crucial to the activity of SPCH, MUTE and FAMA; double Arabidopsis *scrm scrm2* knockout plants fail to produce cells that enter the stomatal lineage (Kanaoka et al., 2008).

There are many hormonal stimuli that directly and/or indirectly regulate stomatal development. For example, the abiotic stress signal abscisic acid (ABA), is known to be an important regulator of both stomatal development and stomatal closure (Chater et al., 2014). However, since our understanding of hormonal regulation in grass stomatal development is limited with respect to our knowledge from Arabidopsis, here we will focus primarily on the core transcription factor-driven stomatal development module. For further information on the hormonal control of stomatal development, see Qi and Torii (2018) or Zoulias et al. (2018).

## Novel and DISTINCt bHLH Functions in Rice and other Grasses

**Figure 3:**
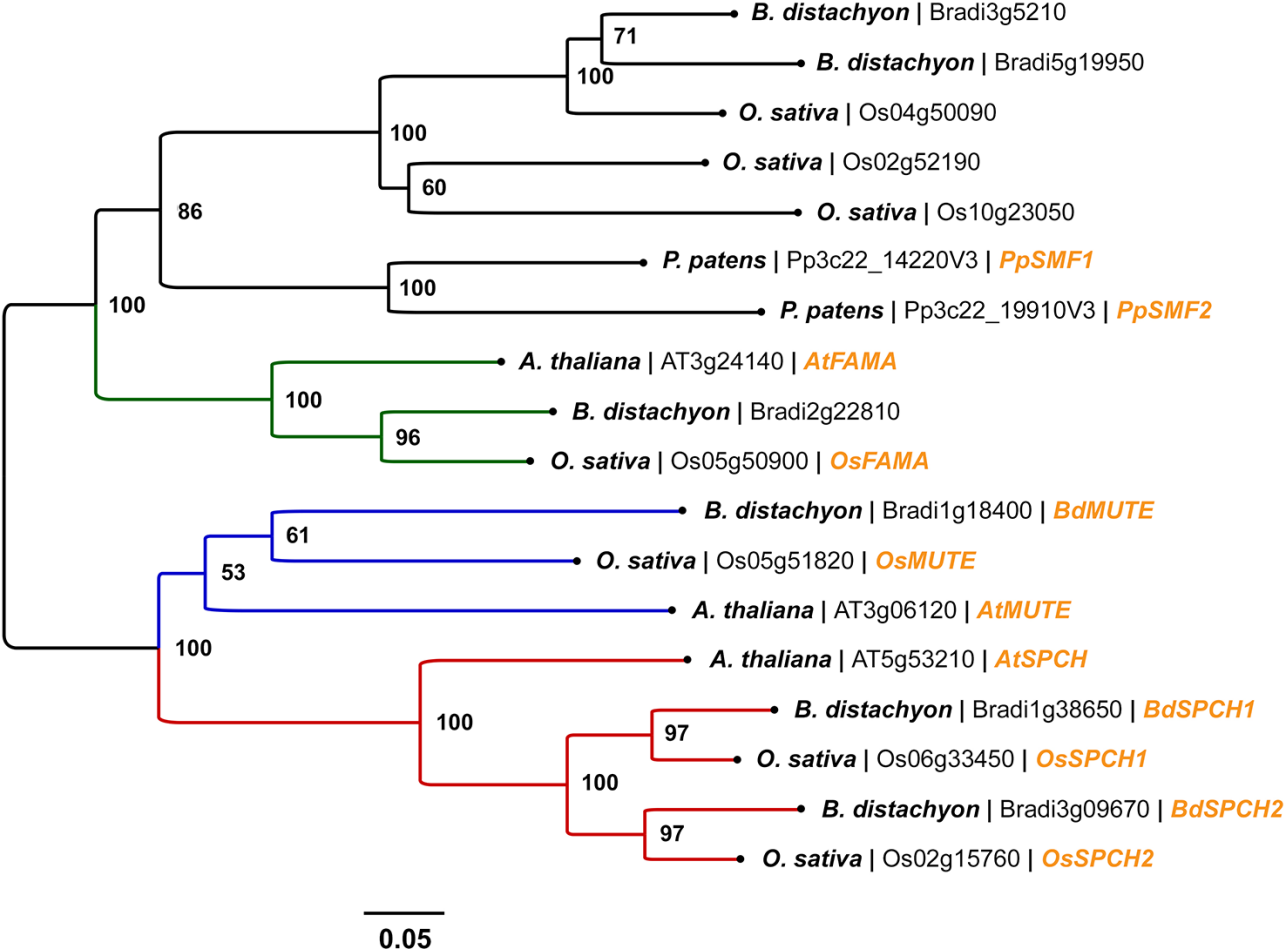
Phylogenetic analysis of SPEECHLESS (SPCH), MUTE, and FAMA peptide sequences in Arabidopsis (*Arabidopsis thaliana*), rice (*Oryza sativa*), and Brachypodium (*Brachypodium distachyon*). Peptide sequences were obtained *via* BLAST searches of the rice and Brachypodium genomes using Phytozome v12. All peptide sequences with expect values (E-values) < 1 x 10^-30^ against AtSPCH, AtMUTE, and AtFAMA were used in the analysis. Stomatal bHLH peptide sequences PpSMF1 and PpSMF2 from the non-vascular moss *Physcomitrella patens* were included for evolutionary context; obtained from Chater et al. (2017). A total of 18 peptide sequences were involved in the phylogenetic analysis. Peptide sequences were aligned using the MUSCLE algorithm (Edgar, 2004). The phylogenetic tree was constructed using the neighbor-joining method (Saitou and Nei, 1987). The optimal tree with the sum of branch length = 4.05347046 is shown. A bootstrap test was performed (1,000 replicates) and the percentage of replicate trees that clustered taxa into the conformation displayed is shown adjacent to the nodes. Evolutionary distances between peptide sequences (in units of amino acid substitutions per site) were calculated using the Poisson correction method (Zuckerkandl and Pauling, 1965). The tree is drawn to scale, with branch lengths proportionate to the evolutionary distances used to infer the tree. All evolutionary analyses were performed in MEGA6. From left to right, species name, gene accession number and gene name (if characterised) are labeled at the branch tips. The clades comprising SPCH, MUTE, and FAMA orthologues are coloured red, blue and green respectively.

Several phylogenetic studies have concluded that *SPCH*, *MUTE*, *FAMA*, *SCRM*, and *SCRM2* are highly conserved across land plants, with *SPCH* undergoing a duplication event in grass species (MacAlister and Bergmann, 2011; Ran et al., 2013; Chater et al., 2017; Qu et al., 2017) (**Figure 3**). Despite the divergence of stomatal morphology and development in grasses (**Figure 1**), bHLH orthologues have now been characterised in both rice (Liu et al., 2009; Wu et al., 2019) and Brachypodium (Raissig et al., 2016), with some discreet differences in functionality detected (**Figure 2**).

Recent research has shown that the two bHLH *SPCH* paralogues — *OsSPCH1* and *OsSPCH2* — both contribute to stomatal development in rice. Despite this, a reduction in GMC number and SD is only observed in single *osspch2* knockout plants (Liu et al., 2009; Wu et al., 2019). Analysis of downstream gene expression in *osspch1* and *osspch2* single mutants support this finding as *OsMUTE* and *OsFAMA* are only downregulated in *osspch2* plants (Wu et al., 2019). However, a more severe effect on development is observed in *osspch1 osspch2* double knockouts where no GMCs or stomata form, suggesting that OsSPCH1 does contribute to early stomatal development in the absence of OsSPCH2 (Wu et al., 2019). Genetic reporter analyses in Brachypodium further emphasize the importance of the grass *SPCH2* paralogue over *SPCH1* (Raissig et al., 2016). A BdSPCH2 signal can be strongly detected from the initiation of stomatal development until SC formation, whereas BdSPCH1 displays a lower overall activity, with the strongest signal occurring after the initial asymmetric division and declining before SC formation begins. Relative to the mild stomatal phenotype of *bdspch1* knockouts, *bdspch2* plants have a drastically reduced SD, consolidating SPCH2 as the main player in initiating stomatal development in grasses (Raissig et al., 2016). Nevertheless, as with rice double knockouts, *bdspch1 bdspch2* plants also fail to form GMCs or stomata, suggesting that in both grass species studied thus far, at least one *SPCH* gene must be present for cells to enter the stomatal lineage (**Figure 2**).

Functioning downstream of SPCH in Arabidopsis, MUTE stops meristemoid amplifying divisions, promotes GMC formation and subsequently promotes the symmetric GMC division which leads to the formation of GC pairs (Pillitteri et al., 2007; Han et al., 2018). Grasses are not thought to possess self-renewing meristemoids, yet by generating *osmute* gene knockout lines, Wu et al. (2019) reported that OsMUTE functions primarily to prevent ectopic divisions of GMCs (**Figure 2**), as GMCs appear to undergo further division(s) before arresting in *osmute* plants. Interestingly, like in rice, ZmMUTE has recently been shown to be responsible for preventing ectopic divisions in maize, with multiple aborted GMCs often found to be stacked within a stomatal file in maize *zmmute* (also termed *bizui2*) mutants (Wang et al., 2019). Ectopic divisions of GMCs often occur in irregular orientations in *osmute* plants and it is unclear whether the stacking phenotype that is observed in maize *zmmute* mutants also occurs in the rice mutants. In both *osmute* and *zmmute*, neither GCs or SCs form, which for *zmmute* plants results in seedling lethality around 14 days post-germination. Taken together, these observations show that if MUTE functionality is compromised it is possible to invoke meristemoid-like activity in grasses. However, as mentioned previously, to date no grass species surveyed has naturally exhibited such a cell type.

In *OsMUTE-*overexpressing plants, Wu et al. (2019) report that all epidermal cells aside from silica and cork cells take on GC-like identity although, upon inspection of the images provided, this suggestion is difficult to interpret as the cells that form appear to be undifferentiated. This is especially apparent when the given examples are contrasted with overexpression studies of *MUTE* in Arabidopsis. In Arabidopsis, ectopic GCs form as part of a stoma and are more clearly GC-like (Pillitteri et al., 2007). To truly determine the identity of the cells generated *via OsMUTE* overexpression — and thus understand more about OsMUTE functionality and governance of cell state — it would be useful to repeat the genetic manipulation of *OsMUTE* in reporter plants that convey GMC, SC, and GC identity.

Whereas neither GCs or SCs form in *osmute* and *zmmute* knockout mutant plants, GC pairs do form in *bdmute* knockout mutant plants, but these GCs are devoid of SCs (Raissig et al., 2017). By generating YFP-BdMUTE reporter lines, it has been demonstrated that the BdMUTE protein is mobile and can promote asymmetric SC entry divisions by entering SMCs from neighboring GMCs (Raissig et al., 2017). Despite the phenotypic discrepancy between *bdmute* mutants and *zmmute* and *osmute* mutants, Wang et al. (2019) showed that ZmMUTE and OsMUTE are also capable of travelling from GMCs to SMCs. Similarly to what was initially described in Brachypodium YFP-BdMUTE reporter lines, YFP-ZmMUTE and YFP-OsMUTE signals are detectable in both GMCs and neighboring SMCs immediately prior to SC-forming divisions in maize and rice, respectively (Raissig et al., 2017; Wang et al., 2019). Yet, unlike *osmute* and *zmmute*, the course of GMC and GC development is less perturbed in *bdmute* knockout plants, and thus MUTE’s involvement in the regulation of GMC cell fate and divisions appears to show some variation amongst different grass species.

OsFAMA function has been analyzed *via* the production of both T-DNA and CRISPR *osfama* mutants, with both methods revealing that OsFAMA acts to promote GC maturation in rice (Liu et al., 2009; Wu et al., 2019) (**Figure 2**). Intriguingly, ectopic GMC divisions (as in *osmute*) and, in some cases, undivided SMC cells are detectable in *osfama* knockout plants (Wu et al., 2019). This implies that FAMA, perhaps in combination with MUTE, contributes to the regulation of SMC asymmetric divisions and GMC symmetric divisions in rice. There are no reports yet that describe how FAMA functions during Brachypodium or maize stomatal development, and so at present it is not possible to compare the function of other grass FAMA orthologues with that of OsFAMA. It will be interesting to learn whether BdFAMA and ZmFAMA also partake in events upstream of GC formation as has been shown in OsFAMA.

Genes with high sequence similarity to Arabidopsis *SCRM* and *SCRM2* have been identified in rice (*Os11g32100* and *Os01g71310* respectively), suggesting that the heterodimerisation of bHLH transcription factors observed in Arabidopsis also occurs in rice (Liu et al., 2009) (**Figure 2**). The proposed protein-protein interactions of these gene products — *OsSCRM* and *OsSCRM2* — with the aforementioned stomatal development genes (OsSPCH1/2, OsMUTE, and OsFAMA) have now been confirmed *in vitro via* bimolecular fluorescent complementation (BiFC) and yeast two-hybrid (Y2H) assays (Wu et al., 2019). Like Arabidopsis SCRM and SCRM2, OsSCRM and OsSCRM2 act redundantly during stomatal development, with double *scrm scrm2* knockouts leading to the absence of all stomatal lineage cell types and stomata in both species (Kanaoka et al., 2008; Wu et al., 2019). Whereas *osscrm* CRISPR knockout mutants fail to produce stomata (and only a few meristemoids form in early development), Arabidopsis T-DNA knockdown plants with little-to-no *SCRM* expression produce a range of stomatal lineage cells (at various aborted stages) as well as stomata (Kanaoka et al., 2008). However, when equivalent *osscrm* T-DNA knockouts are examined in rice (which display a weaker phenotype than CRISPR *osscrm* plants), the function of OsSCRM appears to more closely mirror that of AtSCRM. This is because aborted stomatal lineage cells can be observed throughout the development stages, implying that, together with OsSPCH1/2 in early development, OsSCRM probably associates with OsMUTE and OsFAMA *in vivo* (Wu et al., 2019). In contrast to *scrm* knockouts, stomatal development is unaffected in both *atscrm2* and *osscrm2* knockout mutants, suggesting that these paralogous proteins play a minor role in the development of stomata in their respective species (Kanaoka et al., 2008; Wu et al., 2019).

In Brachypodium, the roles of BdSCRM and BdSCRM2 are more distinct. Both *bdscrm* and *bdscrm2* single mutants produce a seedling lethal phenotype, owing to an inability to produce mature stomata (Raissig et al., 2016). While *bdscrm* plants fail to produce any stomatal lineage cells, entry to the stomatal lineage is not blocked in *bdscrm2* mutants; instead aborted four-cell complexes form which fail to progress to mature stomata (Raissig et al., 2016). This indicates that BdSCRM2 might fulfil a novel function later in development which is independent of BdSCRM (Raissig et al., 2016). It is conceivable that this function is to specifically interact with FAMA and drive the formation of mature stomata. Thus, it will be interesting to learn whether the stomatal phenotype of *bdfama* matches that of *bdscrm2*. Considered across the species discussed, there are clear similarities and some subtle differences in the function and coordination of the bHLH transcription factors; both between the grasses and Arabidopsis and within the grasses themselves.

## Signaling Peptides can Adjust Stomatal Development in Grasses

For plants to adjust their leaf SD, succinct titration of bHLH activity is essential. This is particularly true of SPCH, which has a diverse range of transcriptional targets in Arabidopsis, with chromatin immunoprecipitation-sequencing (ChIP-Seq) experiments identifying over 8,000 genomic binding sites (Lau et al., 2014). To coordinate the activity of SPCH (and other bHLHs) during stomatal development, intra- and extracellular signalling are particularly important and, in Arabidopsis, much research has been conducted into these signalling modules.

Arabidopsis uses apoplastic cysteine-rich signalling peptides called epidermal patterning factors (EPFs) and EPF-like (EPFL) peptides to convey extracellular signals between stomatal lineage cells, mesophyll cells, and cells destined to become pavement cells (Simmons and Bergmann, 2016) (**Figure 2**). EPF2 and EPF1 negatively regulate SD; EPF2 primarily by preventing stomatal lineage entry and meristemoid amplifying divisions (Hara et al., 2009; Hunt and Gray, 2009); EPF1 by restricting meristemoid identity and facilitating the correct orientation of SLGC spacing divisions (Hara et al., 2007; Qi et al., 2017). On the other hand, EPFL9 promotes stomatal development in opposition to EPF2 and EPF1 (Hunt et al., 2010; Sugano et al., 2010). At the plasma membrane, EPF/EPFL signals are perceived by members of the ERECTA family (ERECTA, ER; ERECTA-LIKE1, ERL1; and ERECTA-LIKE2, ERL2) of receptor kinases along with the receptor protein TOO MANY MOUTHS (TMM) (Nadeau and Sack, 2002; Shpak et al., 2005; Lin et al., 2017).The binding of EPF2 to an ER/TMM complex in the presence of somatic embryogenesis receptor kinases (SERKs) promotes signal transduction across the plasma membrane into the cytoplasm; thereby activating the intracellular mitogen-activated protein kinase (MAPK) pathway (Meng et al., 2015). When activated, this pathway culminates in the phosphorylation and subsequent degradation of SPCH (Lampard et al., 2008). EPFL9 acts antagonistically to EPF2 by competing to bind to the ER/TMM complex (Lee et al., 2015). If successful in doing so, EPFL9 can prevent SPCH degradation by blocking activation of the MAPK pathway (Bergmann et al., 2004; Lampard et al., 2008). Rather than an ER/TMM complex, EPF1 preferentially binds to ERL1, which also associates with TMM and, like EPF2, probably competes with EPFL9 for binding (Lee et al., 2015).

Like with the bHLH transcription factors, functional orthologues of EPF/EPFLs are also present in grasses (Tanaka et al., 2013; Caine et al., 2016; Hughes et al., 2017; Yin et al., 2017; Lu et al., 2019) (**Figure 2**). Moreover, phylogenetic analysis also suggests that ERECTA and TMM genes are present, with OsERECTA already shown to be a governor of heat tolerance in rice (Shen et al., 2015). As in Arabidopsis, there are probably two *EPF* genes involved in negatively regulating stomatal development in grasses (Hepworth et al., 2018; Lu et al., 2019). However, rather than one *EPFL9* gene, grasses normally have two; *EPFL9a* and *EPFL9b* (Hepworth et al., 2018; Lu et al., 2019). Using CRISPR-Cas9 and CRISPR-Cpf1 genome editing, Yin et al. (2017) have shown that *OsEPFL9a* plays a major role in regulating rice stomatal development, with *osepfl9a* knockout plants having a ~90% reduction in SD. Conversely, overexpression of *OsEPFL9a* has been shown to moderately increase SD and reduce stomatal size (Mohammed et al, 2019). Interestingly, little-to-no clustering of stomata occurs upon *OsEPFL9a* overexpression in rice, whereas the large increase in density in *EPFL9*-overexpressing Arabidopsis plants is driven by stomatal clustering (Hunt et al., 2010). Despite this, exactly how, where and when *OsEPFL9a* contributes to stomatal lineage progression is still not well understood and requires further study. While no equivalent *OsEPFL9b* knockout or overexpressing rice plants have been generated thus far, recent work in Arabidopsis has shown that *OsEPFL9b* can moderately increase SD when overexpressed (Lu et al., 2019). In contrast to *OsEPFL9a* overexpression, *OsEPFL9b*-overexpressing plants have a tissue-specific clustering phenotype, with stomatal clusters forming in hypocotyls but not rosette leaves (Lu et al., 2019). Nevertheless, based on evidence that is currently available, it seems that *OsEPFL9a* is the dominant player in stomatal development, with *OsEPFL9b* perhaps playing a minor role.

In barley and wheat, Hughes et al. (2017) and Dunn et al. (2019) have shown that overexpression of *HvEPF1* and *TaEPF1B* leads to inhibition of multiple stages of the stomatal lineage, including asymmetric entry divisions, GMC development and the formation of SCs. Overexpression of *HvEPF1* in Arabidopsis produces a similar phenotype to that of Arabidopsis *EPF1* overexpression, whereby meristemoid differentiation is perturbed, resulting in a clustering of small cells around a central meristemoid (Hughes et al., 2017). Given that cells derived from asymmetric entry divisions in grasses are not believed to take on meristemoid-like identity, it is intriguing that HvEPF1 is capable of regulating meristemoid fate in Arabidopsis. This finding, together with findings from wheat, suggests that *EPF1* orthologues probably act relatively early in the stomatal lineage in grasses, but further investigation is required to ascertain exactly where and how these gene products are functioning. Subsequent work by Caine et al. (2019) and Lu et al. (2019), where *OsEPF1* was overexpressed in both rice and Arabidopsis, produced similar results to those in barley and in wheat, with strong overexpression of *OsEPF1* in rice resulting in the majority of protodermal cells failing to enter the stomatal lineage (Caine et al., 2019). As with *OsEPF1*, increasing the expression of *OsEPF2* effectively reduces the SD of both rice and Arabidopsis, with phenotypic similarities to *OsEPF1* overexpression in each instance (Lu et al., 2019). Clearly, assessment of how EPF/EPFLs regulate SD in rice has provided insight to their function, but more research (including generation of *osepf1*, *osepf2* single knockout and *osepf1 osepf2* double knockout plants) is required to enable a more succinct understanding of how rice stomatal development unfolds.

Little is known about how EPF signals are transduced to nuclear-residing bHLH transcription factors during grass stomatal development. However, a recent report has confirmed that like in Arabidopsis, a member of the MAPK pathway, YODA, is involved in regulating stomatal development in Brachypodium (Abrash et al., 2018). Abrash et al. (2018) show that *bdyda1* knockout mutants have a large increase in SD and a severe stomatal clustering phenotype, whereby approximately 86% of stomata are involved in cell clusters. Interestingly, rather than arising as a result of faulty physical asymmetry in cell divisions (as in Arabidopsis *yda* mutants), cell clusters in *bdyda* mutants result from a failure of differentiation and fate reinforcement after asymmetric entry divisions have already occurred (Bergmann et al., 2004; Abrash et al., 2018). Thus, although YDA proteins influence entry to the stomatal lineage in both Brachypodium and Arabidopsis, their roles appear to be distinct. In Arabidopsis, YDA is a pre-division regulator, whereas BdYDA acts post-division to reinforce cell fate in Brachypodium (Abrash et al., 2018).

## Moving Away From the Usual Suspects: Other Players in Rice Stomatal Development

### SCARECROW and SHORTROOT Control Cell File Positioning

Until recently, the positional signals that prompt the specification of grass stomatal cell files remained unclear. Schuler et al.’s (2018) study of rice and maize *SHR* orthologues has shed new light onto this area. In Arabidopsis, SHR interacts with another transcription factor, SCARECROW (SCR), in the shoots to position bundle sheath cells around the developing vasculature (Cui et al., 2014). Given that *(i)* vascular patterning and stomatal patterning are coordinated, *(ii)* stomatal cell files are specified (laterally) adjacent to procambial centres in grasses, and *(iii) OsSHR1* and *OsSCR1* are expressed in stomatal lineage cells, it was proposed that SHR-SCR interactions might contribute to the specification and spacing of stomatal cell files in grasses (Kamiya et al., 2003; Schuler et al., 2018). Indeed, ectopic expression of *ZmSHR1* in the rice epidermis (chosen to avoid potentially silencing another SHR gene, *OsSHR2*) does lead to the formation of extra stomatal cell files positioned further from the leaf veins (Schuler et al., 2018). In line with these findings, double knockout *osshr1 osshr2* rice plants have been shown to have reduced SD, with phenotypes suggesting that OsSHR1/2 act redundantly at multiple points during the stomatal lineage (Wu et al., 2019) (**Figure 2**). Despite these findings suggesting that OsSHR1/2 promote stomatal formation, Wu et al.’s (2019) study does not indicate that overexpression of *OsSHR1* or *OsSHR2* alters the number of stomatal cell files, nor does SD increase when either gene is overexpressed. It is unclear whether this is because of gene silencing (as alluded to above) or whether OsSHR1 or OsSHR2 are genuinely incapable of promoting new stomatal files in rice.

Wu et al. (2019) also investigated the function of OsSCR1 and OsSCR2 during rice stomatal development and found that like OsSHR1/2, OsSCR1 and OsSCR2 positively regulate SD at multiple stages (**Figure 2**). While OsSCR1 has a prominent role, OsSCR2 function is only noticeable in *osscr1 osscr2* double knockouts and not in single knockout *osscr2* mutants. Binding assays have confirmed the existence of OsSHR-OsSCR interactions *in vitro*, suggesting that rice replicates the functional link between SHRs and SCRs that is observed in Arabidopsis. Further analysis of the genomic targets of SHRs and SCRs in grasses might help to clarify exactly how and when they regulate stomatal development and thus may also have implications for the optimisation of SD in rice.

### Cyclins and Cyclin-Dependent Kinases in the Rice Stomatal Lineage

Whereas the rice stomatal bHLHs are thought to invoke and regulate specific cell identities and divisions in the rice stomatal lineage, the precise mitotic control of these divisions has not been well-studied. Across life, specific cyclin-CDK (cyclin-dependent kinase) complexes are known to govern cell cycle transitions (Harashima et al., 2013; Han and Torii, 2019). In the Arabidopsis stomatal lineage, complexes of A2-type cyclins (CYCA2s) and a B-type CDK, CDKB1;1, promote symmetric divisions of GMCs (Boudolf et al., 2009; Vanneste et al., 2011). Undivided GCs are common in triple knockouts of *CYCA2s* (*cyca2;134* and *cyca2;234*) and in *cdkb1;1* mutants, and the nuclear DNA content of cells from the former is double that of normal GCs, suggesting that they are arrested at the G2-to-M phase (Boudolf et al., 2004; Vanneste et al., 2011).

While four CYCA2s are found in Arabidopsis, only one *CYCA2* gene, *OsCYCA2;1*, is present in the rice genome (La et al., 2006; Qu et al., 2018). BiFC assays have shown that OsCYCA2;1 and OsCDKB1;1 (orthologue of Arabidopsis CDKB1;1) form a functional complex in rice (Qu et al., 2018). Yet, in contrast to Arabidopsis, when *OsCYAC2;1* or *OsCDKB1;1* are knocked down, GMC divisions are uninterrupted (Qu et al., 2018). Intriguingly, fewer asymmetric entry divisions are observed in stomatal cell files in *OsCYCA2;1*-RNAi plants, leading to an overall reduction in SD (Qu et al., 2018). Nevertheless, both *OsCYCA2;1* and *OsCDKB1;1* can rescue the defective phenotypes of Arabidopsis *cyca2* and *cdkb1* mutants respectively (Qu et al., 2018). Thus, the functions of CYCA2s and CDKB1;1 must be somewhat conserved between rice and Arabidopsis despite the divergence in the timing of their activity (Qu et al., 2018).

Connections between the core bHLH module and cell-cycle regulators in the Arabidopsis stomatal lineage are beginning to be identified (Xie et al., 2010; Lau et al., 2014; Adrian et al., 2015; Han et al., 2018). In the early lineage, *CYCD3s* are upregulated in response to *SPCH* expression (Adrian et al., 2015), with *CYCD3;1* targeted directly by SPCH (Lau et al., 2014). Later in development, inducers of symmetric GMC divisions — *CYCD5;1*, *CYCA2s*, and *CDKB1;1* — are directly upregulated by MUTE; a recent finding that has implicated MUTE as a governor of the GMC symmetric division (Han et al., 2018). FAMA and FLP/MYB88 are responsible for negatively regulating this symmetric division by ensuring that GCs do not undergo further divisions (Lai et al., 2005; Lee et al., 2014). Whereas MUTE enhances the expression of *CDKB1;1*, FLP represses its expression by binding to a *cis*-regulatory region in its promoter sequence (Xie et al., 2010). A single orthologue of *FLP/MYB88*, *OsFLP*, is present in the rice genome (Wu et al., 2019). Akin to its role in Arabidopsis, OsFLP appears to be involved in the regulation of symmetric divisions in rice, as knocking out its expression leads to abnormal division of GMCs (Wu et al., 2019) (**Figure 2**). However, as described earlier, CDKB1;1 does not influence the GMC-GC transition in rice, so the genomic targets of OsMUTE and OsFLP must have diverged to some extent. Thus, exactly how the functions of other cyclins and CDKs are programmed in the rice stomatal lineage must now be determined. Furthermore, by investigating the cyclin-CDK-mediated regulation of asymmetric SC entry divisions, insight will be gained as to how the regulatory machinery of the plant cell-cycle has been adjusted in grasses to accommodate these unique cell divisions.

## Manipulating Gas Exchange in Rice by Altering Stomatal Density

### Decreasing Stomatal Density Improves WUE and Drought Tolerance

Our extensive knowledge of stomatal development in Arabidopsis is gradually being translated into rice and other grasses. Now, by harnessing this knowledge, efforts are being made to alter the stomatal development of rice to create crops that might be better suited to future climate conditions. Decreasing the number of stomata on the leaves of rice could maintain its productivity in the future hotter, drier climate (Caine et al., 2019). Reducing SD in Arabidopsis, maize, and barley demonstrably improves WUE and/or drought tolerance (Hepworth et al., 2015; Liu et al., 2015; Hughes et al., 2017). However, until Caine et al.’s (2019) study, it was unclear whether this finding could be replicated in rice given the potentially negative impact of constraining the transpiration rate of such a water-intensive crop (Hoekstra and Chapagain, 2006). To test this hypothesis, Caine et al. (2019) engineered a rice cultivar (IR64) to overexpress *OsEPF1*, creating plants with up to an 88% reduction in stomata density (relative to wildtype SD). Direct examinations of whole-plant water use have shown that *OsEPF1*-overexpressing (*OsEPF1oe*) plants consume around 40% less water than control plants over equivalent periods, due primarily to reductions in *g_s_* (Caine et al., 2019).

In future predictions of the climate, plants will be faced with a dilemma: close stomata to save water or keep them open to stay cool (Chaves et al., 2016). Whereas both elevated atmospheric CO_2_ or reduced water availability trigger stomatal closure in plants, exposure to increased temperatures could lead to lethal overheating or photoinhibition if transpirational cooling is not maintained (Bertolino et al., 2019). By this logic, plants engineered to have fewer stomata should be more susceptible to heat-stress (Urban et al., 2017). However, thermal imaging of *OsEPF1oe* and comparable control plants subjected to droughted conditions reveals a heightened capacity for evaporative cooling in *OsEPF1oe* plants during late-stage drought. Essentially, *OsEPF1oe* plants are able to restrict transpiration during the early stages of drought, meaning more water is available for maintenance of evaporative cooling later in the drought (Caine et al., 2019). Moreover, under well-watered conditions at elevated temperature, *OsEPF1oe* plants can increase the aperture of their stomata, thereby mitigating the potentially detrimental effect of having fewer stomata when conditions are more favorable (Caine et al., 2019).

Although results have not been replicated in the field, during laboratory-simulated drought treatments *OsEPF1oe* grain yields are at least equivalent to controls (Caine et al., 2019). Interestingly, when drought is introduced during flowering (after 88 days of growth), lines with moderate stomatal reductions (*OsEPF1oeW*) outperform both control plants and those with more severe reductions (*OsEPF1oeS*). In these plants, both grain yield and above-ground biomass are increased, suggesting that subtle adjustments to SD might be beneficial during drought at the flowering stage (Caine et al., 2019). After the same treatment, the 1,000 grain weight of all *OsEPF1oe* lines is significantly higher than that of controls (Caine et al., 2019). Exposure to high temperatures during grain filling is known to reduce the content of starch molecules and storage proteins in rice grains (Lin et al., 2010). Therefore, the observed maintenance of grain weight in *OsEPF1oe* lines in droughted conditions might be due to prolonged transpirational cooling of the heat-sensitive flowers (Morita et al., 2016).

### Can Increases to Stomatal Density Improve Photosynthetic Efficiency?

It has been shown that changes to stomatal size and density can be correlated with *g_s_*, and so it follows that genetic manipulation of SD has the potential to increase photosynthetic gas exchange (Franks et al., 2012). However, as pointed out by Harrison et al. (2019), such a coupling between SD and gas exchange does not always exist in practice. Nevertheless, Arabidopsis *EPFL9*-overexpressing plants (with ~600% increased SD) show enhanced *A* at both ambient and elevated CO_2_, probably due to a much increased *g_s_* (Tanaka et al., 2013). This is despite the occurrence of stomatal clustering, a trait that has been shown to negatively impact photosynthetic performance (Dow et al., 2014). In rice plants that overexpress *OsEPFL9a*, gas exchange (both *A* and *g*_s_) is unchanged, although this may be a signature of the smaller increase in SD (~20%–30%) in these plants relative to Arabidopsis *EPFL9-*overexpressing plants (Mohammed et al., 2019). A similarly modest increase in SD in rice has been generated *via* the overexpression of a maize *SHORTROOT* gene, *ZmSHR1* (Schuler et al., 2018). Like with *OsEPFL9a* overexpression, higher SD in *ZmSHR1*-overexpressing plants does not lead to an enhancement of *A* or *g_s_* (Schuler et al., 2018). For *OsEPFL9a* overexpression, the lack of increase in either parameter may be explained by a concurrent reduction in stomatal size (Mohammed et al., 2019). Perhaps if changes to the stomatal number were more significant — as in Tanaka et al. (2013) — the compensatory effect of reduced stomatal size in *OsEPFL9a*-overexpressing plants might have less of an impact in preventing *g_s_* from increasing.

As discussed previously, manipulating SD will create a trade-off between the uptake of CO_2_ and the control of transpirational water flow. Indeed, in Arabidopsis, enhancement of *A* in Tanaka et al.’s (2013) *AtEPFL9*-overexpressing plants is not translated to biomass gains. This is likely due to the plants being more prone to water loss, as transgenic plants with greater SD have significantly greater transpiration rates (Tanaka et al., 2013). Relative to Arabidopsis and other plants, rice might be more able to afford an increased rate of water loss, as the majority (~75%) of production is sourced from irrigated lowland systems (Zhao et al., 2011). Moreover, amongst lowland rice varieties, *A* is correlated with SD when plants are grown in flooded soils (Tsunoda and Fukoshima, 1986). In fact, the SD and *g_s_* of 3 high-yielding rice cultivars (IR72, Takanari and LYPJ) are significantly higher than the corresponding average values from 69 lower-yielding accessions (Ohsumi et al., 2007).

### Linking SD With Stomatal Size and Physiology to Improve WUE

Genetic manipulation of SD leads to a fixed change in the number of stomata that form per unit area in the epidermis. The stomata of Arabidopsis SD mutants (of multiple genotypic backgrounds) retain the capacity to open and close and changes in SD are often accompanied by inverse changes to stomatal size; i.e., reductions in SD are linked to increases in stomatal size and vice versa (Doheny-Adams et al., 2012). However, this size-density response is not consistently seen in grasses. For both barley and the IR64 rice cultivar (subspecies indica) the opposite response is observed, whereby reductions in SD result in co-reductions in stomata size (Hughes et al., 2017; Caine et al., 2019). In the Nipponbare rice cultivar (subspecies japonica), reduced SD leads to increased stomatal size and increased SD leads to reduced size; responses similar to those previously observed in Arabidopsis (Mohammed et al., 2019). Thus, within different subspecies of rice, the stomatal size responses to genetic manipulation of SD are different. Why this occurs is unclear, although there is evidence in grasses that smaller stomata can improve WUE by opening and closing quicker in response to environmental fluctuations (McAusland et al., 2016; Lawson and Vialet-Chabrand, 2019). For both subspecies, it would be interesting to assess how the stomata of low-SD *OsEPF1oe* plants perform under fluctuating conditions to ascertain the impact that size and density alterations have on the “speed” of stomatal movements and WUE.

By targeting the mechanisms that govern guard cell ion transportation across the plasma membrane and tonoplast, it is possible to effectively alter stomatal opening and closing in response to both abiotic and biotic stress (Huang et al., 2009; Daszkowska-Golec and Szarejko, 2013; Li et al., 2017; Lawson and Vialet-Chabrand, 2019) and to enhance plant biomass accumulation (Papanatsiou et al., 2019). Because the SCs of grasses are thought to both enable a swifter transport of ions and osmolytes to GCs and to lend a mechanical advantage to their stomatal movements, grass stomatal complexes are viewed as being more responsive than other stomatal morphologies (Franks and Farquhar, 2006; Cai et al., 2017; Nunes et al., 2019). Indeed, the stomata of *bdmute* plants (which are devoid of SCs) respond more slowly to changes in light intensity (Raissig et al., 2017). However, to date no genetic manipulations that enhance the speed of grass stomata have been reported, and it is therefore not possible to compare the potential water savings that could be achieved by low-SD rice with those of rice manipulated to have enhanced stomatal closure. To address this unknown, future efforts to manipulate rice stomata should aim to target short-term stomatal responses, perhaps individually and in combination with SD, so that a more complete picture of the control of WUE in grass plants can be developed.

## Conclusion

Rice is the world’s most important human food crop and yields must be protected against future climate instabilities. As the “gatekeepers” of transpiration and carbon uptake, stomata represent an obvious target to improve *A* or water retention in rice (Lawson and Blatt, 2014). Although more detailed examinations of rice stomatal development will be required to match our understanding of stomatal development in Arabidopsis, the advances to our understanding of grass stomatal development discussed here have enabled high- and low-SD rice plants to be developed (Schuler et al., 2018; Caine et al., 2019; Mohammed et al., 2019). Contrary to expectation, rice plants with increased SD do not exhibit corresponding increases in *A* (Schuler et al., 2018; Mohammed et al., 2019), although this could be due to altered stomatal size and or aperture size. Thus, rice plants with more substantial increases in SD will be required to test the efficacy (or lack thereof) of targeting stomatal development for the enhancement of either *A* or evaporative cooling. On the other hand, given that freshwater insecurities and exposure to drought will likely become increasingly prevalent in areas of rice cultivation (Kang et al., 2009; Quentin Grafton, 2017), rice crops that use water more efficiently might be of greater importance in the future climate. This will be especially important in Africa where, as a result of water limitations, rainfed upland rice production is steadily increasing, despite this method being particularly susceptible to drought (Saito et al., 2018). Promisingly, *OsEPF1oe* lines with decreased SD exhibit improved water conservation and tolerance to drought without yield penalties (Caine et al., 2019). While this development is encouraging, these findings have not yet been reproduced in the field, and so it remains to be seen whether real-world fluctuations in environmental variables will influence the performance of these plants that have thus far only been tested under laboratory conditions.

## Author Contributions

CB, RC, and JG wrote the article and CB prepared the figures. All authors checked and approved the article before publication.

## Funding

RC and JG acknowledge the BBSRC and Newton fund for the financial support. RC also acknowledges the University of Sheffield QR GCRF fellowship (Research England institutional allocation) for the fellows.

## Conflict of Interest

The authors declare that the research was conducted in the absence of any commercial or financial relationships that could be construed as a potential conflict of interest.
